# Family Partnership in Hypnosis to Improve Mechanical Ventilation Weaning in an ICU Patient With High Cervical Spinal Cord Injury—A Case Report

**DOI:** 10.1111/nicc.70299

**Published:** 2025-12-17

**Authors:** Christine Bernard, Margrit Ascher, François Vaast, Chiara Martinez, Kevin Chalard, Pierre‐François Perrigault

**Affiliations:** ^1^ Department of Anaesthesia and Critical Care Medicine Gui de Chauliac CHU Montpellier, Univ Montpellier Montpellier France

**Keywords:** family‐centred care, humanising critical care, hypnosis, quadriplegia, ventilator weaning

## Abstract

Quadriplegia often leads to severe respiratory impairment, accompanied by distressing symptoms such as dyspnoea and breathlessness. Ventilator weaning remains a challenge in the acute phase of high cervical spinal cord injury. We present the case of a 27‐year‐old man with traumatic C5–C6 quadriplegia. His clinical course was complicated by recurrent respiratory issues and a prolonged ventilator weaning process. Medical hypnosis was introduced as a complementary strategy to support ventilator weaning. A nurse anaesthetist certified in medical hypnosis performed the intervention. As sedation limited direct communication, the patient's mother participated in creating a personalised ‘safe place’ script based on family memories and the patient's personal resources. Hypnosis helped reduce anxiety and dyspnoea, contributing to the success of spontaneous breathing trials. It also strengthened the therapeutic alliance by involving the family. This case study underscores the potential role of hypnosis in critical care, particularly when developed in collaboration with family members. Family involvement promotes a more personalised and humanised approach to care. In this case, family partnership promoted respiratory autonomy and facilitated ventilator weaning.

## 1 Introduction

Respiratory failure is common in patients with quadriplegia. Spinal cord injuries above C6 can compromise diaphragm function as well as accessory respiratory muscles. Additionnal complications—including airway obstruction, bronchial hyperreactivity and reduced thoracic compliance—may further impair ventilation [1]. In this context, ventilator weaning is often complex, prolonged and distressing for patients and their families.

Hypnosis which is defined as a ‘modified state of consciousness’ that modulates brain networks is increasingly recognised as a non‐pharmacological intervention in intensive care [2]. Although hypnosis is well established in pain management [3], it may also alleviate the emotional aspect of dyspnoea [4]. Like pain, dyspnoea involves sensory discomfort and emotional responses [5]. By reducing the unpleasantness of breathlessness and mitigating anxiety, hypnosis may facilitate the transition to spontaneous breathing.

Hypnosis requires a conscious and willing participant who can contribute personal imagery and sensory cues. In this case, however, sedation and the patient's inability to speak restricted access to his subjective experience. To preserve the integrity of the process, the patient's mother provided sensory and biographical elements, that allowed for the creation of a personalised ‘safe place’ script that remained faithful to the patient's identity.

Thus, families may serve as mediators of the patient's inner world by contributing memories, values and personal imagery. This case study illustrates how family‐partnered hypnosis supported ventilator weaning in a young man with quadriplegia, highlighting both its clinical potential and its role in advancing family‐centred care.

## 2 Clinical Findings

### 2.1 Patient Information

Alex (pseudonym), a 27‐year‐old man, had no significant medical history apart from a prior ICU admission in 2021 for head trauma which left no long‐term sequelae. On 28 February 2022, he sustained a traumatic neck injury while playing rugby, resulting in immediate quadriplegia. He was airlifted to the neurological ICU at Montpellier University Hospital.

An MRI revealed a C5–C6 fracture with an associated spinal cord injury. Upon admission, a neurological examination showed complete tetraplegia with flaccid paralysis and loss of motor and sensory function below the C5 level. His ASIA score was 8 of 100.

He underwent cervical spine osteosynthesis on the day of the injury, followed by a tracheostomy on March 9.

Deep sedation was frequently required during his ICU stay due to recurrent respiratory decompensation and ventilator asynchrony. Sedation consisted of continuous infusions of propofol, midazolam, ketamine and sufentanil, along with norepinephrine for hemodynamic support. After a pulmonary embolism was diagnosed on March 6, the patient began anticoagulation therapy with unfractionated heparin. This therapy continued until April 14, when it was transitioned to subcutaneous enoxaparin (Lovenox).

The patient's ICU course was complicated by several significant respiratory events, including right lung atelectasis unresponsive to prone positioning or antibiotics; a pulmonary embolism; and hypoxic cardiac arrest, from which he recovered quickly. He also developed compressive left pneumothorax. Overall, he required 64 days of invasive mechanical ventilation, including 46 days under sedation (Table 1).

**TABLE 1:** Respiratory events during hospitalisation.

	Sedation Y/N	Mechanical ventilation mode/FiO_2_	Respiratory events	Therapeutic interventions	Weaning: behavorial observations
*28/02*	Y	VC‐AC/25%		Sedated during 1 week	
*03/03*	Y	VC‐AC/30%	Hypoxemia	Changed for a non‐armoured endotracheal tube Bronchoalveolar washing	
*06/03*	Y	VC‐AC/70%	Pulmonary embolism Ventilator‐associated pneumoniae	Antibiotics Heparin	
*09/03*	Y	VC‐AC/50%	Tracheotomised		
*13/03*	Y	VC‐AC/60%	Bibasal atelectasis	Deeped sedated	
*16/03*	Y	SPN/40%	Right basal atelectasis		
*20/03*	Y	VC‐AC/60%	Right atelectasis	Deeped sedated Bronchoalveolar washing	
*22/03*	Y	VC‐AC/60%	Pneumothorax Right atelectasis	Drainage	
*25/03*	Y		Right atelectasis	Bronchoalveolar washing	
*29/03*	Y	VC‐AC/50%	Right atelectasis	Bronchoalveolar washing	
*30/03*	Y	VC‐AC/50%	Right atelectasis	Bronchoalveolar washing Prone position (with the surgeon's consent)	
*03/04*	Y	VC‐AC/50%	Right atelectasis	Bronchoalveolar washing Prone position	
*07/04*	Y	VC‐AC/40%	Right atelectasis Alveolar derecruitment when SPN hypoxia		SPN Impossible
*11/04*	N	VC‐AC/30%	Right atelectasis	Positioning Verticalisation	
*14/04*	N	VC‐AC/30%		Change for compact home adapted respirator	
*17/04*	N	VC‐AC/30%	Right atelectasis	Positioning Verticalisation	
*19/04*	N	VC‐AC 30%		Verticalisation Abdominal belt	Hypoxia after 2 h SPN
*25/04*	N	VC‐AC/30%			SPN during the day Dyspnoea
*28/04*	N	SPN/30%			SBT using hypnosis
*29/04*	N	SPN/30%		Change of cannula with fenestred inner cannula	
*03/05*	N	SPN/30%		Positioning Verticalisation	SBT
*05/05*	N	0		Speaking valve	
*11/05*	N	0		Decannulation	
*15/05*	N	0	Right atelectasis	Cannula replacement	SBT during the day SPN/30% during the night
*16/05*	N	0		ICU discharge	SBT during the day SPN/30% during the night

*Note:* Mechanical ventilation with Dräger Evita V800.

Abbreviations: 0: spontaneous breathing; FiO_2_, fraction of inspired oxygen; ICU, intensive care unit; SBT, spontaneous breathing trial; SPN: spontaneous‐assisted mode (SPN‐CPAP/PS: spontaneous‐continuous positive airway pressure/pressure support); VC‐AC, volume control–assist control.

## 3 Interventions

### 3.1 Conventional Management

Weaning followed the best‐established practices for high cervical spinal cord injuries. These practices included abdominal binding and favouring the supine position during spontaneous breathing trials [6]. Despite repeated attempts over the first 6 weeks, all trials failed. On April 14, his ICU ventilator was replaced with a compact, home‐adapted device to facilitate the transition to rehabilitation.

Due to persistent challenges and growing family distress, medical hypnosis was introduced as a complementary intervention. A nurse anaesthetist formally trained in medical hypnosis conducted the sessions. A preliminary review of theoretical and clinical littérature suggested that hypnosis could support weaning patients with high cervical spinal cord injuries by modulating the sensory and emotional dimensions of dyspnoea. In this case, hypnosis was used to promote respiratory autonomy by addressing the physiological and psychological aspects of care.

Four hypnosis sessions were conducted during the weaning process, each aligned with clinical opportunities for spontaneous breathing trials. While there are no current standardised recommendations regarding the optimal number of sessions, Fernandes et al. [7] recently demonstrate that a brief intervention consisting of five hypnosis sessions focusing on the emotional regulation of dyspnoea, when combined with pulmonary rehabilitation, produces sustained benefits for at least 6 months. These findings support the use of short, repeated hypnosis sessions to reinforce emotional coping and respiratory control mechanisms during critical care weaning.

The potential benefits were explained, and his relatives were immediately convinced of its value. As Alex could not speak, a preparatory session was held with his mother to develop the ‘safe place’ imagery. She played an active role in creating this scene without conducting hypnosis herself. She provided vivid sensory details, including the smell of wood, the sound of birdsong and the coolness of the nearby river. These elements formed the basis of a personalised hypnosis script tailored to Alex's identity and history.

### 3.2 Hypnosis Sessions (Table 2)

**TABLE 2:** Hypnosis sessions description.

	Anaesthesiologist hypnotherapist nurse action	Aim of the session	Hypnosis session details	Patient's clinical state
*07/04*	Preparation of the safe place with his mother	Positive projection of Alex's helpers	Conversational hypnosis	Sedated, VC‐AC
*11/04*	Relaxation	Experiment hypnosis effects, deep relaxation	Safe place and suggestions 20 min	Lighted sedated, VC‐AC
*22/04*	First SBT + hypnosis support	Break the anxious part of dyspnoea to try SBT, aware of his resources	Safe place and suggestions 20 min	Non‐sedated, VC‐AC and SPN
*28/04*	Second SBT + hypnosis support	Break the anxious part of dyspnoea to try SBT, aware of his resources	Safe place and suggestions 20 min	Non‐sedated, SPN
*29/04*	SBT during verticalisation + Hypnosis support	At patient's request to break dyspnoea	Safe place and suggestions 20 min	Non‐sedated, SPN
*29/04*	Teach self‐hypnosis	Hypnotherapist weaning	Post‐hypnotic suggestions	Non‐sedated, SPN

Abbreviations: SBT, spontaneous breathing trial; SPN, spontaneous‐assisted mode (SPN‐CPAP/PS: spontaneous‐continuous positive airway pressure/pressure support); VC‐AC, volume control—assist control.

April 22: First spontaneous breathing trial (SBT) using a T‐tube under hypnosis. The session lasted 15 min but it was stopped due to abdominal distention and atelectasis, though gas exchange remained stable. Alex reported a comfort level of 8 of 10.

From April 23 to 28, three additional SBTs under hypnosis were successful without oxygen desaturation.

On April 29, placement of a fenestrated Shiley cannula improved coughing and phonation. The patient requested that the procedure be performed under hypnosis.

On May 11, complete decannulation was achieved.

On May 15, recannulation was required due to recurrent right‐sided atelectasis. However, Alex was fully weaned from mechanical ventilation at discharge.

## 4 Discussion

Managing patients with quadriplegia requires a long‐term, multidisciplinary approach that extends from intensive care to rehabilitation. Although respiratory function may improve over time due to reduced inflammation, decreased chest wall stiffness and neural adaptation [1], dyspnoea remains highly prevalent, affecting up to 77% of patients with high‐level spinal cord injuries [8].

Previous studies indicate that weaning from the ventilator after a high cervical spinal cord injury typically requires 8–12 weeks [9]. In this case, the overall weaning duration of approximately 10 weeks was consistent with the expected trajectory. However, once respiratory instability had resolved, the patient progressed rapidly and tolerated spontaneous breathing trials particularly well during hypnosis‐assisted sessions, reporting improved comfort and reduced dyspnoea.

### 4.1 The Role of Hypnosis in Dyspnoea Modulation

Hypnosis acts as a complementary intervention, reducing dyspnoea and interrupting the cycle in which respiratory discomfort increases anxiety worsening breathlessness.

Unlike bottom‐up models of sensory processing, where afferent respiratory signals are translated into conscious perception, hypnosis predominantly operates via top‐down modulation [10]. Through mental imagery and personal representations, higher order cortical networks influence the interpretation of sensory input. This allows patients to reframe and reduce the perceived unpleasantness of dyspnoea.

Neuroimaging research supports this mechanism. Although most studies focus on pain, dyspnoea has a similar dual sensory‐affective structure [5]. Hypnosis primarily modulates the affective component. Rainville et al. [11] demonstrated that hypnotic modulation of pain unpleasantness selectively alters anterior cingulate cortex activity without altering somatosensory cortex activation. Similarly, Faymonville et al. [12] demonstrated that hypnosis enhances functional modulation within networks governing sensory, affective, cognitive and behavioural processing. These findings support a top‐down mechanism in which hypnosis reshapes the emotional significance of sensory stimuli.

### 4.2 Implications for Nursing Practice

From a nursing perspective, hypnosis can be a practical therapeutic tool, not just an extension of the therapeutic relationship. Training nurses in hypnosis increases its accessibility and facilitates its integration into routine ICU practice. As a communication technique, hypnosis can reduce distress, personalise care and strengthen the therapeutic alliance, even when verbal communication is limited.

In our unit, the ‘Resource Wall’ enables families to share photos and personal notes about patients' preferences and daily lives. This helps preserve the patient's biography during periods of prolonged sedation, ensuring that the patient is approached as a person with a rich life history rather than an anonymous body. For caregivers, the wall serves as a bridge between critical illness and lived experience, informing relational and therapeutic approaches.

A notable aspect of this case was the mother's contribution to the hypnotic script. In cases where communication is challenging, family members can play a crucial role in providing essential biographical and sensory information. They can do so by using familiar language and shared experiences to guide the development of personalised interventions that would otherwise be inaccessible. Family‐partnered hypnosis is in line with the broader movement toward family‐centred ICU care, promoting not only symptom relief but also resilience, therapeutic alliances and family agency. In this case, the mother's involvement proved to be pivotal, as she provided the sensory and emotional elements that anchored the hypnosis experience.

## 5 Strengths and Limitations

This is a single case, which limits generalisability. The highly personalised hypnosis intervention may not be replicable without family participation or in other cultural contexts. Additionally, it is difficult to isolate the effect of hypnosis on ventilator weaning due to concurrent medical care and natural recovery processes. Future research should evaluate structured hypnosis protocols in ICU care.

## 6 Patient Perspective

Alex and his family were informed that hypnosis was being used on an exploratory basis.

From the beginning, Alex was highly motivated. After his stay in the ICU, he reported having no memory of experiencing respiratory discomfort during the four hypnosis‐assisted spontaneous breathing trials. He did not remember the first session, but he clearly recalled the others. He described the sessions as profoundly helpful for ventilator weaning and noted that he experienced no respiratory difficulty while under hypnosis. He vividly recalled sensory impressions of the imagined scene, including the smell of barbecue, the voices and laughter of friends and physical sensations such as relaxation and tingling, which made the experience feel real and reassuring.

One year later, he was decannulated and using an electric wheelchair. He shared a video capturing a symbolic moment: his wheelchair was strapped onto a quad trailer, and he returned—proudly—to the shack.

## 7 Conclusion

This case study emphasises the valuable role of hypnosis in ventilator weaning for patients with quadriplegia. Involving family members in creating hypnotic scripts provides access to patients' resources, supports respiratory autonomy and makes ICU care more humane.

## Funding

The authors have nothing to report.

## Ethics Statement

Written informed consent was obtained from the patient. ‘Alex’ is a pseudonym used to protect privacy.

## Conflicts of Interest

The authors declare no conflicts of interest.

## Data Availability

The data that support the findings of this study are available from the corresponding author upon reasonable request.
